# *Legionella pneumophila* subverts the antioxidant defenses of its amoeba host *Acanthamoeba castellanii*

**DOI:** 10.1016/j.crmicr.2024.100338

**Published:** 2025-01-07

**Authors:** Alban Hay, Willy Aucher, Romain Pigeault, Joanne Bertaux, Alexandre Crépin, Quentin Blancart Remaury, Yann Héchard, Ascel Samba-Louaka, Romain Villéger

**Affiliations:** a Université de Poitiers, UMR CNRS 7267, Ecologie et Biologie des Interactions, France; b Université de Poitiers, UMR CNRS 7285, Institut de Chimie des Milieux et Matériaux de Poitiers, France

**Keywords:** Acanthamoeba castellanii, Antioxidant defenses, Host-pathogen interaction, Legionella pneumophila, Reactive oxygen species, T4SS dependent

## Abstract

*Legionella pneumophila*, the causative agent of Legionnaires' disease, interacts in the environment with free-living amoebae that serve as replicative niches for the bacteria. Among these amoebae, *Acanthamoeba castellanii* is a natural host in water networks and a model commonly used to study the interaction between *L. pneumophila* and its host. However, certain crucial aspects of this interaction remain unclear. One such aspect is the role of oxidative stress, with studies focusing on reactive oxygen species (ROS) production by the host and putting less emphasis on the involvement of the host's antioxidant defenses during the infectious process. In this study, we propose to examine the consequences of infection with *L. pneumophila* wild-type or with an isogenic Δ*dotA* mutant strain, which is unable to replicate intracellularly, on *A. castellanii*. For this purpose, we looked at the host ROS levels, host antioxidant defense transcripts, and metabolites linked to the amoeba's antioxidant defenses. It is known that *L. pneumophila* WT can block the activation of NADPH oxidase as soon as it enters the macrophage and suppress ROS production compared to Δ*dotA* mutant strain. In addition, it has been shown in macrophages that *L. pneumophila* WT decreases ROS at 24 h p.i.; here we confirm this result in amoebae and suggest that this decrease could be partly explained by *L. pneumophila* differentially regulated host antioxidant defense transcripts at 6 h p.i.. We also explored the metabolome of *A. castellanii* infected or not with *L. pneumophila*. Among the 617 metabolites identified, four with reduced abundances during infection may be involved in antioxidant responses. This study suggests that *L. pneumophila* could hijack the host's antioxidant defenses during its replication to maintain a reduced level of ROS.

## Highlights

• *Legionella pneumophila* induced an upregulation of antioxidant defense transcripts in *Acanthamoeba castellanii* at 6 h post-infection (p.i.), resulting in decreased ROS quantity at 24 h p.i..
• We identified 97 intracellular metabolites disrupted during the infection of *A. castellanii* by *L. pneumophila* in a TSS4-dependent manner.
• Four metabolites in *A. castellanii* exhibited a reduced abundance following infection by *L. pneumophila*, potentially contributing to the maintenance of an antioxidant state in the host.
• Pre-exposure of amoebae to pro-oxidizing molecules may facilitate bacterial multiplication at 24 h p.i..

## Graphical abstract

**Figure:**
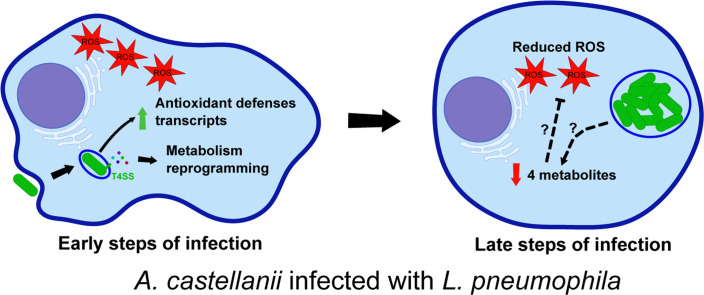

## 1 Introduction

*Legionella pneumophila* is responsible for a severe form of pneumonia called Legionnaires’ disease. According to the World Health Organization, Europe, Australia, and the USA have about 10–15 cases detected per million people annually (World Health Organization, 2024). The disease is only accidentally transmitted to humans by inhalation of droplets contaminated with bacteria from air-conditioning systems, water pipes, cooling towers, etc (Kanarek et al., 2022). In the lungs, the bacteria infect the pulmonary alveolar macrophages, resist phagocytosis, and cause symptoms ranging from a simple flu-like illness (Pontiac fever) to severe pneumonia (Legionnaires' disease). Despite the ability of *L. pneumophila* to infect, replicate in, and exit from macrophages, its adaptations to resist phagocytosis by human cells are unlikely to be fixed by this dead-end interaction (Leenheer et al., 2023). This resistance to phagocytosis most likely stems from previous interactions with the bacterium's natural hosts, such as amoebae, which provide both a multiplication site and a “training camp” for the bacteria (Price et al., 2024; Samba-Louaka et al., 2019). The Dot/Icm type IV secretion system (T4SS) allows the bacterium to deliver more than 330 effectors in the host cell acting on many signaling and metabolic pathways to its benefits (Mondino et al., 2020). The infection cycle shares similarities between amoebae and macrophages from adhesion, inhibition of lysosome fusion, and the setting up of an intracellular replicative niche called *Legionella*-containing vacuole (LCV) to evasion mainly by causing the host lysis (Escoll et al., 2013). Nevertheless, some aspects of this interaction remain unclear. This is the case of oxidative stress in the host, when the balance between reactive oxygen species (ROS) quantity and antioxidant defense is compromised (Li et al., 2021).

ROS production is an antimicrobial mechanism occurring during phagocytosis (Dupré-Crochet et al., 2013). One of the most important roles of ROS in host-pathogen interactions is their involvement in bacteria clearance under oxidative stress. Indeed, ROS are small molecules that can diffuse across the membrane of the bacteria, and damage bacterial nucleic acids, proteins, and lipids, impacting bacterial growth (Fisher, 2009). Although neutrophils kill *L. pneumophila* using ROS from NADPH oxidase (Price et al., 2021; Ziltener et al., 2016), the bacterium suppresses ROS production in infected U937 cells via its T4SS due to the failure of NADPH oxidase activation through inhibition of p47phox recruitment to phagosomes (Harada et al., 2007). Another study has shown that *L. pneumophila* decreases ROS in RAW 264.7 macrophages at 24 h p.i. assuming that this would constitute a potential virulence mechanism, but without speculating on the origin of this reduction (Kajiwara et al., 2018). Nevertheless, to our knowledge, no study has demonstrated the inhibition of NADPH oxidase recruitment or reduction of ROS by *L. pneumophila* in amoebae.

*A. castellanii* contains the full set of components for both thioredoxin (Trx) and glutathione (GSH) systems, as well as other well-known antioxidant defense enzymes, such as superoxide dismutase (SOD) and catalases (Clarke et al., 2013). The Trx and GSH systems use NADPH to reduce hydrogen peroxide (H_2_O_2_) or disulfide bonds and maintain a reduced state in the cell. With their redox properties, the Trx and GSH systems can act in many cellular processes, such as cell survival, proliferation and apoptosis (Aquilano et al., 2014; Lee et al., 2013). The GSH system is composed of glutathione (GSH, L-γ-glutamyl-L-cysteinyl-glycine), glutathione peroxidase (Gpx), glutathione reductase (GR), and glutaredoxin (Grx). The Trx system is made up of thioredoxin (Trx), thioredoxin reductase (TrxR), and peroxiredoxin (Prx) (Staerck et al., 2017). It's also interesting to note the structural similarities between these two systems, one being a potential backup for each other (Holmgren, 1989; Holmgren et al., 2005; Marty et al., 2009). Indeed, a study has shown that oxidative stress in *A. castellanii* induced by H_2_O_2_ led to significant stimulation of the thioredoxin system, while, treatment with auranofin, a gold salt known to inhibit TrxR activity, led to transcriptional activation of the GSH system (Köhsler et al., 2022). This backup option was already shown for human Grxs, which can reduce Trx1 and Trx2 production when TrxR is inhibited (Du et al., 2012; Zhang et al., 2014).

A specificity of *A. castellanii* is the presence of two types of TrxRs, a high-molecular-weight TrxR (TrxR-L) and a low-molecular-weight TrxR (TrxR-S) (Leitsch et al., 2021). TrxR-L is typically found in higher eukaryotes, mammals, and humans, while TrxR-S is usually found in bacteria, fungi, plants, and lower eukaryotes. It has been shown that they are both present in the cytosol and that only TrxR-S transcripts and proteins are both present in greater quantities under oxidative stress conditions (Leitsch et al., 2021). In an earlier work, we observed that *L. pneumophila,* in the late-stage of infection, triggered *A. castellanii* proteome modifications including an increase in the production of antioxidant enzymes (Hay et al., 2023).

Based on our previous results and the current knowledge of antioxidant defense used by *A. castellanii* under oxidative stress, in this work we explored the involvement of oxidative stress in the interaction between *A. castellanii* and *L. pneumophila*. For this purpose, we investigated the gene expression of enzymes associated with oxidative stress in *A. castellanii* during infection by *L. pneumophila* wild-type (WT) and its isogenic mutant lacking the *dotA* gene (Δ*dotA*) and measured intracellular ROS production in the amoeba*.* Subsequently, we conducted an analysis of the intracellular metabolome of *A. castellanii* during infection by *L. pneumophila* WT or the Δ*dotA* mutant to identify metabolic pathways involved in *L. pneumophila*-induced oxidative stress in a *dotA*-dependent manner.

## 2 Materials and methods

### 2.1 Amoeba and bacteria growth conditions

*A. castellanii* ATCC 30010 was cultured in Peptone Yeast Glucose medium (PYG, 2 % proteose peptone, 0.1 % yeast extract, 0.1 M glucose, 4 mM MgSO_4_, 0.4 mM CaCl_2_, 0.1 % sodium citrate dihydrate, 0.05 mM Fe(NH_4_)_2_(SO_4_)_2_ 6H_2_O, 2.5 mM NaH_2_PO_3_, 2.5 mM K_2_HPO_3_, pH 6.5) at 30°C.

*L. pneumophila* Paris CIP 107629T, hereafter designated as Lp WT (Cazalet et al., 2004) and its isogenic Δ*dotA* mutant, hereafter designated as Lp Δ*dotA* (resistant to kanamycin at 15 μg/mL) which is a gift of Carmen Buchrieser (Gomez-Valero et al., 2014) were propagated on Buffered Charcoal Yeast Extract (BCYE, 1 % ACES, 1 % yeast extract, 0.2 % charcoal, 1.5 % agar, 0.025 % Iron (III) pyrophosphate, 0.04 % L-cysteine, 0.1 % alpha-ketoglutarate, pH 6.9) agar plate at 37°C for three days. Then, they were inoculated at the optical density at 600 nm (OD600) of 0.1 in Buffered Yeast Extract (BYE) and grown at 37°C under agitation at 180 rpm for 24 h to reach an OD600 around four. Lp WT or Δ*dotA* carrying the pMMB207-Km14-GFPc plasmid (Tiaden et al., 2007) designated as Lp WT or Δ*dotA* GFP, were generated according to the protocol previously described (Mengue et al., 2016). These transformants were cultivated in BYE supplemented with chloramphenicol (5 μg/mL).

### 2.2 Infection of *A. castellanii* with *L. pneumophila*

Amoebae in PYG were seeded in six or twelve well plates, in 2 or 1 mL of PYG respectively, and incubated for 1 h at 30°C without shaking for adhesion. Then, the growth medium was discarded and replaced with a bacterial suspension of *L. pneumophila* in Page's Amoeba Saline solution (PAS, 4 mM MgSO_4_, 0.4 M CaCl_2_, 0.1 % sodium citrate dehydrate, 2.5 mM NaH_2_PO_3_, 2.5 mM K_2_HPO_3_, pH 6.5) buffer at a multiplicity of infection (MOI) of 20. The infection was synchronized by centrifugation (500×*g*, 10 min at room temperature) followed by a 1 h incubation at 30°C. The medium was replaced by PYG containing gentamicin (100 μg/mL) for 1 h at 30°C then by PYG alone. Plates were incubated at 30°C up to the time required for the experiment. Cell viability of infected and non-infected amoebae was assessed by staining them with Trypan Blue: samples were diluted in a 1:1 ratio with Trypan Blue (0.4 % Solution) and cells were enumerated on a FastRead 102® (Biosigma) counting chamber using a light microscope.

### 2.3 Amoebae mechanical lysis for bacterial enumeration

Intracellular bacteria were counted after amoebae lysis by flow cytometry as described in section 2.8. Amoebae suspensions were transferred to 2 mL microcentrifuge tubes with socket screw caps (VWR®, Radnor, PA, USA), then mechanically lysed using a FastPrep-24™ 5G (MP Biomedicals, Irvine, CA, USA) with the following settings: three steps of 30 sec at speed 5.0 m/s, with 5 min incubation on ice between each cycle.

### 2.4 RNA isolation and cDNA synthesis

In 2 mL of PYG, 1×10^6^ amoebae/well were seeded in six wells-plate. They were infected or not as mentioned before (section 2.2) with Lp WT or Lp Δ*dotA* or exposed during 2 h at 750 µM H_2_O_2_ as a positive control. At 2, 6, and 24 h post-infection (p.i.), 3 wells were pooled, and RNA was extracted using the RNeasy Mini kit (QIAGEN, Germantown, MD, USA), following the supplier recommendations. To remove genomic DNA, samples were treated with TURBO DNA-free™ Kit (Invitrogen, Waltham, MA, USA) following provider instructions in the provider protocol. The RNA concentration and purity were determined using a NanoDrop™ One spectrophotometer (ThermoFisher Scientific, Wilmington, DE, USA). Only samples with a 260/280 ratio between 1.8 and 2.0 were used for subsequent analyses. 300 ng of RNA per reaction was used for cDNA synthesis using the GoScript™ ReverseTranscription System (Promega, Madison, WI, USA). All cDNA samples were diluted to 10 ng/µL using qPCR-grade water and stored at 80°C for further processing.

### 2.5 Quantitative PCR

Quantitative PCR (qPCR) was performed on a LightCycler® 480 thermal cycler (Roche, Rotkreuz, Switzerland) using the Takyon™ No ROX SYBR 2X MasterMix blue dTTP kit (Eurogentec, Seraing, Belgium) according to the supplier's recommendations. The primers used are presented in Table 1. Eight microliters of reaction mix containing Takyon mix 1X with 0.3 μM of forward/reverse primers diluted in qPCR-grade water were added to 2 μL of cDNA. The qPCR cycling program was as follows: 95°C for 5 min followed by 40 cycles at 95°C for 10 s, 56 or 58°C for 10 s, 72°C for 10 s. The gene expression level was calculated based on the threshold cycle (CT) normalized to the CT of the reference gene, here a portion of the 18S rRNA (Köhsler et al., 2020). The relative expression data were calculated by the 2^−ΔΔCT^ method (Livak and Schmittgen, 2001) and the experiment was performed three times independently for each sample.

**Table 1:** Primers used for RT-qPCR in this study.

Primer	Primer sequence (5′-3′)	Amplicon length (bp)	Average Tm (°C)	Amplification efficiency (%)	Source (Genbank® accession number)	Targeted gene	Thyb (°C)
18ST4	F: CCCAGATCGTTTACCGTGAA	147	60.5	94.13	Köhsler *et al.* 2020	*18S rRNA gene*	58
	R: TAAATATTAATGCCCCCAACTATCC		59.5				
Gpx	F: GAACTCACTGCCGAGGACAA	174	59.97	92.88	XM_004335951	*glutathione peroxidase Hyr1*	58
	R: GGGGAAGCCCACAATCTCAA		59.96				
GR	F: CGACACTCTCTACAACAACC	149	59.5	103.39	Köhsler *et al.* 2022 (XM_004338198)	*glutathione-disulfide reductase*	56
	R: CTTCTCGTCACGCTTGGAC		61.6				
Grx	F: GGAGATGAGAGCGTTCAG	184	56.3	96.15	Köhsler *et al.* 2022 (XM_004339719)	*glutaredoxin, putative*	58
	R: CTCTTGGCCTGCATCTCG		58.4				
GS	F: TGGGAGCTCTACGGAGACAA	105	59.96	87.38	XM_004337577	*glutathione synthetase*	56
	R: CCTGTCCCACAGCGTGTATT		60.04				
Prx	F: CTCTCGTGGCCGATCTTACC	196	59.97	86.24	XM_004333592	*peroxiredoxin 2*	58
	R: TCGTCAACGTACTGGAAGGC		60.04				
SOD	F: TACCCCGCTGGAGAACAAGA	121	60.54	92.68	XM_004341390	*manganese and iron superoxide dismutase*	56
	R: CATGCAGTCCCAGTAGAACGA		59.80				
Trx	F: GCCATCGAGAAGATGAGCCA	185	59.89	98.85	XM_004335461	*thioredoxin-1, putative*	56
	R: GTGATGCCCTCAGCGATCTT		60.18				
TrxR-S	F: CTCTCGAACCCCAAGATC	182	56.3	88.02	Köhsler *et al.* 2022 (XM_004351629)	*thioredoxin-disulfide reductase*	58
	R: CACCTGACCATTCAGGAAC		57.5				
TrxR-L	F: TGCTACGCCAAGCTCATCTG	192	60.46	94.46	XM_004353581	*thioredoxin reductase 1, cytoplasmic, putative*	56
	R: AACTTCGAGCGTCGTGAACT		59.97				

### 2.6 Analysis of *A. castellanii* intracellular metabolome by UHPLC-HRMS

In 2 mL of PYG, 1×10^6^ amoebae/well were seeded in three wells of six wells-plate. As mentioned before, amoebae were infected with Lp WT or Δ*dotA* or grown in PYG alone (negative control). At 6 h and 24 h p.i., PYG was removed and replaced by 1 mL of PAS. The contents of the three wells were scraped off and pooled. The number of intracellular bacteria was assessed by the colony-forming unit (CFU) determination: 1 mL of infected amoebic suspension from each condition was directly lysed as described above (section 2.3). The suspensions were serially diluted and cultured on BCYE agar plates 72 h at 37°C for viable bacteria enumeration.

The remaining 2 mL of each sample were centrifuged 5 min at 2000×*g* and the supernatants were discarded. The pellets were suspended in 1 mL of Acetonitrile/Methanol/Water mix (2/2/1) (Riedel-de Haënand™, Seelzen, Germany) and transferred into 2 mL microcentrifuge tubes with socket screw caps (VWR®, Radnor, PA, USA). The freeze-thaw method was performed by immersing samples three times in liquid nitrogen then mechanical lysis was performed as described above (section 2.3). Finally, a centrifugation step was performed at 14000×*g* for 10 min at 4°C. The volume of supernatant corresponding to 1×10^6^ amoebae was collected for each sample. In addition, external quality control sample (QC) was prepared by pooling 5 µL aliquots from all samples. All samples were kept at -80°C until analysis.

Ultra-high-pressure liquid Chromatography High-Resolution Mass Spectrometry (UHPLC-HRMS) analysis were conducted using Thermo Scientific Ultimate 3000 pumps interfaced with a Q-Exactive (Hybrid Quadrupole-Orbitrap Mass Spectrometer manufactured by Thermo Fisher Scientific). Compound ionization was achieved through Heated ElectroSpray Ionization (HESI). The Xcalibur data system (Thermo Fisher Scientific) controlled UHPLC mobile phases and HRMS functions. LTQ Velos ESI Positive Ion Calibration Solution (Pierce Biotechnology Inc., Rockford, IL, USA) was injected daily for accurate mass calibration. Compounds underwent separation on a Hydrophilic Interaction Liquid Chromatography (HILIC) column. Specifically, an Acquity UPLC® BEH amide analytical column (150 mm×2.1 mm, 1.7 μm; Waters Corporation, Milford, MA, USA) fitted with an Acquity UPLC® BEH Amide Vanguard guard column (2.1 mm×5 mm, Waters Corporation) was utilized. Ten microliters of samples were injected. Elution was carried out at a constant flow rate of 300 μL.min^−1^ at 30°C. Acetonitrile/water (85/15) with 0.15 % formic acid served as mobile phase A, while water with 0.15 % formic acid and 10 mM ammonium formate served as mobile phase B. The gradient started with 100 % A, remaining constant for 0.5 minutes, then transitioning to 95 % A in 2.30 minutes. Over the subsequent 10 minutes, the percentage of A was reduced to 80 % and reached 70 % in 4 minutes. Subsequently, it decreased to 60 % A over 2 minutes and remained constant for another 2 minutes. The column was then reconditioned for 1 minute with 100 % A. The pooled QC sample was run as every 5th sample throughout the whole LC-HRMS experiment.

For the MS method, the electrospray voltage was set at 2.8 kV, the capillary temperature maintained at 120°C, and the heater temperature at 300°C. Sheath, sweep, and auxiliary gas flow rates were set at 40, 0, and 30, respectively (arbitrary units). The Q-Exactive operated in positive mode at a resolution level of 70,000 for Full MS and 17,500 for dd-MS2, covering a mass range of 70–1050 amu.

LC-HRMS raw data were processed using Compound Discoverer™ 3.3 software (Thermo Fisher Scientific, San Jose, CA, USA). The software followed a workflow that performed peak detection, grouping, retention time correction, QC correction of intensities and potential identification (Fig S2). The parameters used for the workflow are presented in Supplementary_data_1. Features annotation was performed using mzCloud (a library derived from Orbitrap data), mass lists (an in-house developed repository of suspect metabolites), and ChemSpider (a comprehensive library incorporating multiple compound databases). The “WT6_4” replicate was excluded from the analysis due to insufficient quality MS signal for this sample. To highlight potential discrimination between sample groups, multivariate statistical models were built. Principal Component Analysis (PCA) analysis and partial least-squares discriminant analysis (PLS-DA) were performed using Metaboanalyst 5.0 software (https://www.metaboanalyst.ca, Canada) (Pang et al., 2021). Hierarchical clustering heatmaps were performed using MetaboAnalyst 5.0 software with auto-scaled features and Euclidean distance measurement with the Ward clustering method. The identified features and their peak areas were analyzed using the web-based extension (https://www.metaboanalyst.ca, Canada). Only metabolites identified in two of three used databases (*i.e.* mzCloud, mass list, and ChemSpider), with variable importance in projection (VIP) score > 1, p-value < 0.05 from an FDR T-test, and biologically relevant were represented.

### 2.7 Flow cytometry

In 1 mL of PYG, 1×10^5^ amoebae/well were seeded in 12 wells-plate. Amoebae were infected with Lp WT GFP as mentioned before or were pre-treated for 3 h with 250 µM H_2_O_2_ or 5 µM auranofin. Amoebae were scraped off then half was directly analyzed, and the other half was lysed as described above before analysis. When necessary, suspensions were stained with 5 µM CM-H_2_DCFDA (Invitrogen™, Carlsbad, CA, USA) for 30 min at 30°C. This chloromethyl derivative of 2',7'-dichlorodihydrofluorescein diacetate is oxidized by intracellular ROS and becomes fluorescent. Measurements were performed with a CytoFLEX flow cytometer (Beckman Coulter, Brea, CA, USA) equipped with a blue diode laser (excitation 488 nm) managed by CytExpert 2.0.0.153 software (Beckman Coulter). For the CM-H_2_DCFDA and GFP signal, the 525/40 nm emission filter was used.

### 2.8 Fluorescent microscopy

In 1 mL of PYG, 5×10^4^ amoebae/well were seeded in 24 wells-plate with a glass bottom. They were stained with 1 µM CM-H_2_DCFDA (Invitrogen™) for 30 min at 30°C then washed with PAS. Amoebae exposed 30 min to 250 µM H_2_O_2_ served as a positive control. Images were recorded at 6 and 24 h p.i. using an Axio Observer A1 coupled with an apotome (Carl Zeiss, Marly-le-Roi, France) for automated three-dimensional acquisition. The microscope was equipped with a mercury lamp, and both Set 44 and Set 49 Zeiss Filters. Images were analyzed with Zen software (Zeiss, Oberkochen, Bade-Wurtemberg, Germany) and the integrated density signal of H_2_DCFDA was measured using ImageJ software (three independent experiments with at least four technical replicates).

### 2.9 Statistical analysis

Analyses were carried out using R (v. 4.4.4). The Shapiro-Wilk test for normality and Kruskal–Wallis or one-way ANOVA tests with Dunn's and Tukey's post hoc tests for pairwise comparison respectively were performed using package rstatix (Kassambara, 2023). The influence of treatments on amoebae number, ROS-linked median signal of H_2_DCFDA fluorescence, infection prevalence, GFP median signal and bacteria number within amoebae were analyzed using lmer or glmer (package: lme4 (Bates et al., 2015)) according to whether the error structure was normally (numbers of amoebae and bacteria within amoebae, ROS and GFP signal) or binomially distributed (infection prevalence). Technical replicates nested within biological replicates were used as random factors in the models. For each model, we checked either the homoscedasticity and distribution of the residuals or the overdispersion using the DHARMa package (Hartig and Lohse, 2022). Maximal models were simplified by eliminating non-significant terms to establish a minimal model (Crawley, 2013). The significance of the explanatory variables was established using a likelihood ratio test (which is approximately distributed as a Chi-square distribution (Bolker, 2008)). A posteriori contrasts were carried out by aggregating factor levels together and by testing the fit of the simplified model using a likelihood ratio test (Crawley, 2013). Statistical significance of supplementary data was determined using multiple comparisons with a Kruskal–Wallis test on ranks followed by a Dunn's post hoc test or a non-parametric Mann–Whitney U test (GraphPad Prism 8.0.1; GraphPad Software, San Diego, CA, USA). All data are averages of three independent experiments and error bars represent the standard error of the mean (±SEM). .

## 3 Results and discussion

### 3.1 *L. pneumophila* decreases the ROS quantity in *A. castellanii* at 24 h post-infection

**Fig. 1:**
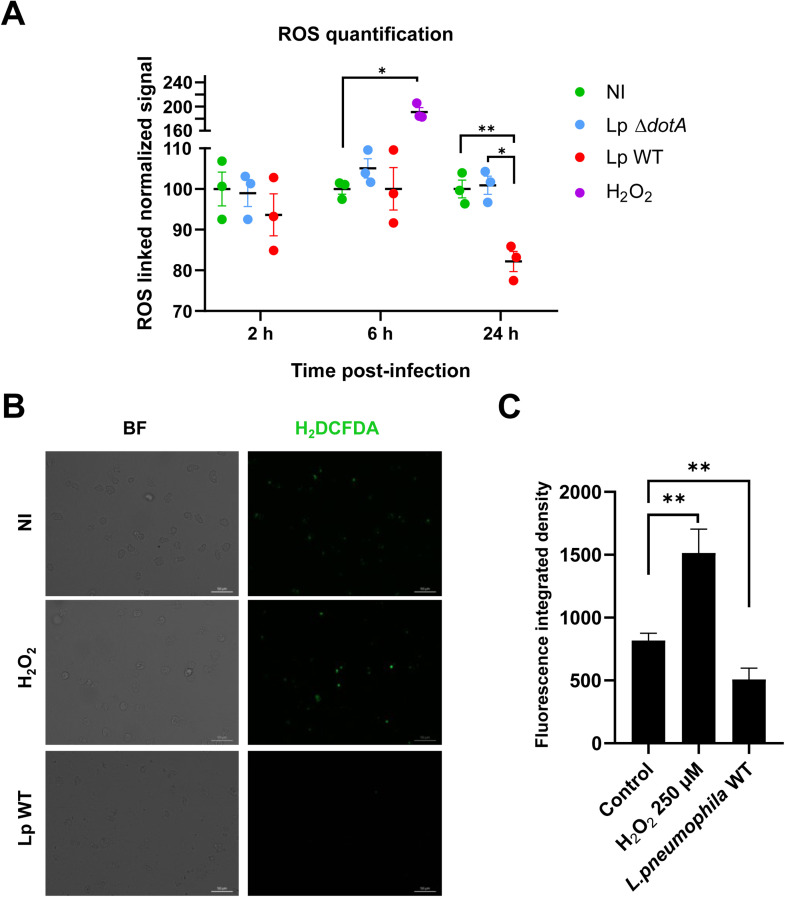
***Legionella* infection decreases the intracellular level of ROS at 24 h p.i..** (A) The diagram shows the percentage normalized to non-infected amoebae (NI) levels of ROS in non-infected amoebae (NI) compared to infected with *L. pneumophila* wild-type (Lp WT) or *dotA* deficient (Lp Δ*dotA*) at 2, 6 or 24 h p.i. or treated with 750 µM H*_2_*O_2_ (H_2_O_2_) as a positive control. The normalized levels of ROS are represented in percentage based on data obtained from flow cytometry. They have been represented as mean ± SEM of three independent experiments done in triplicate. (B) Representative micrographs of non-infected amoebae (NI), treated with 750 µM H*_2_*O_2_ (H_2_O_2_), and Lp WT using fluorescence microscopy, at 24 h p.i. CM-H_2_DCFDA staining is green on the right (brightfield left column). BF: Bright Field. (C) Fluorescence integrated density of CM-H_2_DCFDA fluorescent signal of non-infected amoebae (NI), treated with 250 µM H*_2_*O_2_ (H_2_O_2_), and infected by Lp WT at 24 h p.i. (from three independent experiments with at least four technical replicates). ** p < 0.01.

*L. pneumophila* has already been shown to reduce macrophage ROS at 24 h p.i. (Kajiwara et al., 2018). Thus, fluorescent labeling kinetics using H_2_DCFDA to measure ROS levels in amoebae were performed in triplicate across three independent experiments (Fig. 1A). As a positive control, the amoebae challenged with H_2_O_2_ showed a significant increase of about 90% of the median ROS-related fluorescence signal compared to uninfected amoebae. No significant difference was observed at 2 h and 6 h p.i. between groups. Our results suggest a tendency toward increased ROS production at 6 hours post-infection with the mutant strain lacking a functional T4SS (Δ*dotA*), a trend not observed with the wild-type strain (Fig. 1A). This supposes that *L. pneumophila* might inhibit ROS production in the amoebic host *via* its T4SS at early stages of infection, consistent with findings in macrophages reported by Harada *et al.* (2007). However, a 18% significant decrease in ROS signal in amoebae infected with Lp WT at 24 h was reported compared to other conditions. This result was confirmed by microscopic observations (Fig 1B) associated with quantification of fluorescence integrated density of these observations at 24 h p.i. (Fig. 1C). Indeed, a significant difference was noted between non infected amoebae and amoebae infected with *L. pneumophila* WT with a decrease of 16.5% of fluorescence integrated density in the latter case, which closely aligns with the reduction observed by flow cytometry. *Escherichia coli* K1 uses Lpp, a lipoprotein, to decrease neutrophils ROS production (Zhang et al., 2023). *Anaplasma phagocytophilum* scavenges ROS (Carlyon et al., 2004) and *Francisella tularensis* directly prevents NADPH oxidase assembly (McCaffrey and Allen, 2006).

Our results showing a decrease in ROS are in line with what has been shown in macrophages infected with *L. pneumophila* WT compared to infection with the Δ*dotA* mutant at 24 h p.i. (Kajiwara et al., 2018). Nevertheless, the origin of this decrease remained unclear, so we decided to focus on the host antioxidant defense transcript.

### 3.2 *L. pneumophila* upregulated several genes involved in amoeba antioxidant defense

A recent proteomic analysis has shown that *L. pneumophila* can increase abundance of 15 proteins belonging to GO term associated with antioxidant activity (GO:0016209) (Hay et al., 2023). Based on these results, we have selected nine genes. Four of them encode enzymes of the glutathione system: glutathione peroxidase (Gpx), glutathione reductase (GR), glutaredoxin (Grx), and glutathione synthetase (GS). We also selected four genes encoding enzymes of the thioredoxin system: low-molecular-weight thioredoxin reductase (TrxR-S), thioredoxin (Trx), high-molecular-weight thioredoxin reductase (TrxR-L), and peroxiredoxin (Prx). A final gene encoding a superoxide dismutase (SOD) was also tested. In this article, for clarity, the enzyme names will be abbreviated and used as the associated gene names in italics. The full names of the genes and their Genbank® accession numbers are given in Table 1.

Fold changes in RNA expression of the host genes studied at 2 h, 6 h, and 24 h after infection by Lp WT or Lp Δ*dotA* compared with non-infected (NI) amoebae are shown in Fig. 1. Amoebae challenged with 750 μM H_2_O_2_ for 2 h were used as positive control of induced oxidative stress. The positive control was extracted concurrently with the 6 h p.i. samples. Regardless of the gene studied, no significant difference in expression was observed at 2 h p.i. in amoebae infected with Lp WT or Lp Δ*dotA*. However, a transient effect was observed at 6 h p.i. with a return to the starting point at 24 h p.i.

**Fig. 2:**
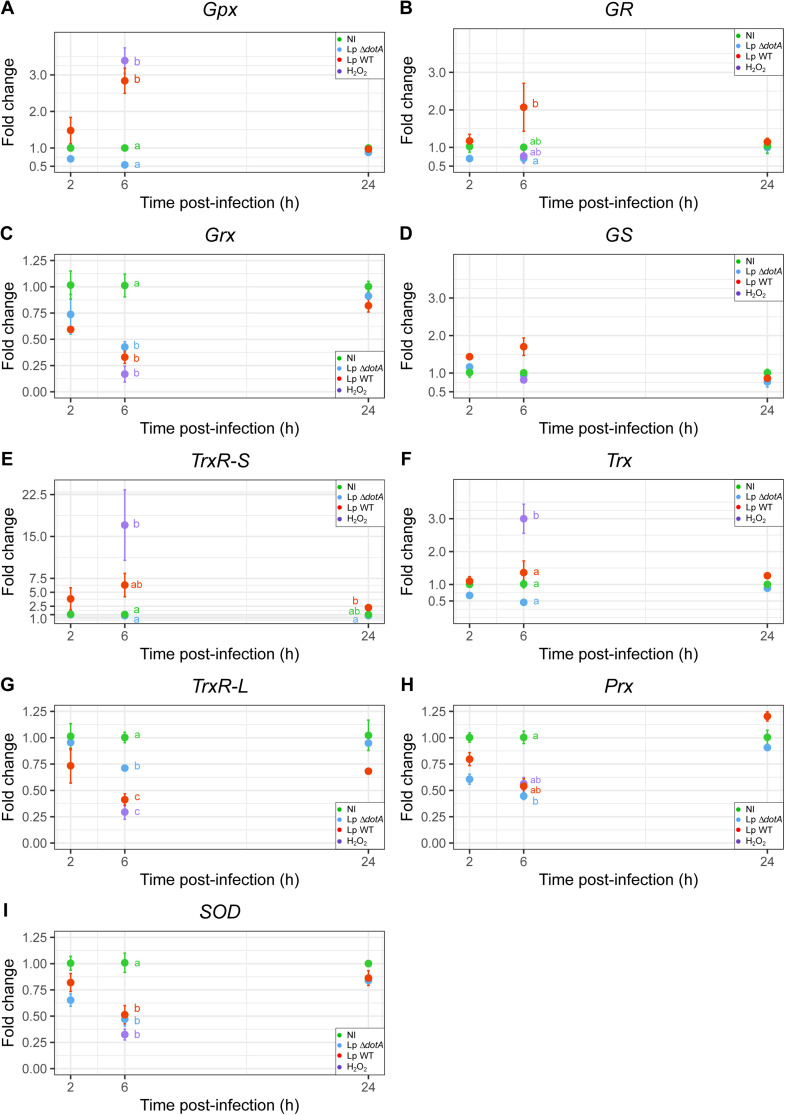
***L. pneumophila* regulates the expression of *A. castellanii* antioxidant genes.** Gene expressions of non-infected amoebae (NI) were compared to cells infected for 2 h, 6 h and 24 h with *L. pneumophila* wild-type (Lp WT) or *L. pneumophila dotA* deficient (Lp Δ*dotA*), or non-infected amoebae challenged with 750 μM H_2_O_2_ for 2 h as positive control. The graphs present genes fold-change studied using RT-qPCR of: (A) Glutathione peroxidase Hyr1 gene (*gpx*), (B) Glutathione-disulfide reductase gene (*gr*), (C) Glutaredoxin, putative gene (*grx*), (D) Glutathione synthetase gene (*gs*), (E) Thioredoxin-disulfide reductase gene (*trxR-S*), (F) Thioredoxin-1, putative gene (*trx*), (G) Thioredoxin reductase 1, cytoplasmic, putative gene (*trxR-L*), (H) Peroxiredoxin 2 gene (*prx*), (I) Manganese and iron superoxide dismutase gene (*sod*). The y-axis indicates RNA levels fold change compared to non-infected controls. Error bars show the standard error of the mean (SEM). All values were obtained from three biological replicates done in duplicate corresponding two-well qPCR plates. At each time, two points not connected by the same letter are significantly different *i.e.* p-value < 0.05, no letter for a particular time means no significant difference.

*Gpx* and *gr* presented a similar profile regarding mRNA levels (Fig 2A and Fig 2B respectively), with 2.8 and 2.1-fold increase in expression in Lp WT-infected amoebae at 6 h p.i. respectively when compared to NI amoebae. A transcriptomic study of *A. castellanii* infected with *L. pneumophila* WT or Δ*dotA* at 16 h p.i. has shown that one *gr* gene was slightly up-regulated (Li et al., 2020). Gpx was also shown to play a crucial role in infection, as the mitochondrial *Coxiella* effector protein F from *Coxiella burnetii* regulates the location of a Gpx to protect the host cell from ROS-induced cell death (Loterio et al., 2023). We can assume that the up regulation of *gr* and *gpx* by Lp WT is likely aimed at preventing the death of its host and ensuring its survival by reducing H_2_O_2_ with glutathione.

Intriguingly, the H_2_O_2_-challenged amoebae *gr* expression showed no difference from uninfected amoebae contrary to *gpx* expression. It could be due to a non-preferential involvement of the glutathione system in *A. castellanii* to manage oxidative stress induced by H_2_O_2_ (Köhsler et al., 2022). However, a study on *A. castellanii* exposed 24 h to 50 µM H_2_O_2_ has shown a slight significant increase in the amount of GR protein, involving the possible use of this system for prolonged exposure (Motavallihaghi et al., 2022). This study has also reported a strong increase in Gpx protein quantity.

These results could suggest a specific induction of the host *gpx* and *gr* by *L. pneumophila* WT *via* its T4SS at 6 h p.i. Indeed, no difference in expression was noticed for amoebae infected with *L. pneumophila* Δ*dotA* although bacteria were still associated with amoebae at this time after infection (Fig S1A).

For *grx* at 6 h p.i. (Fig. 2C), expression in amoebae infected with Lp WT or Lp Δ*dotA,* or exposed to H_2_O_2_ was significantly downregulated compared to uninfected amoebae. In *A. castellanii*, the corresponding enzyme was assumed as not being involved in oxidative defense (Köhsler et al., 2022). Regarding the decrease of *grx* transcripts during infection by Lp WT in our study, the *grx* tested here was probably involved in other cellular processes than antioxidant defenses. Nevertheless, among genes identified in the *A. castellanii* genome (Clarke et al., 2013), two glutaredoxins transcripts (including a putative one) were increased in amoebae infected with Lp WT compared with Lp Δ*dotA* (Li et al., 2020). This could indicate the bacteria's use of host glutaredoxin, possibly to reduce ROS-oxidized cysteine residues and maintain an antioxidant environment favorable to its multiplication.

Finally, the *gs* profile regarding mRNA levels (Fig. 2D) revealed no significant difference in expression between all conditions even if we could notice a general aspect like *gr* expression. Indeed, a 1.7-fold increase in expression is observed at 6 h p.i. in amoebae infected with Lp WT. It could reflect the promotion of host GSH synthesis which is going in the same direction as the increases of *gr* and *gpx* transcription. In addition, it was already reported that some auxotrophic bacteria to GSH, like *Streptococcus pyogenes* or *Haemophilus influenzae* hijack host glutathione for their antioxidant defense system (Brouwer et al., 2022; Vergauwen et al., 2010).

For the Trx system, a significant increase of both *trxR-S* (Fig 2E) and *trx* (Fig 2F) expression under oxidative stress was noted with fold change of 17.0 and 3.0 respectively. The same results were already shown in previous studies with *A. castellanii* (Leitsch et al., 2021) and *C. albicans* (Enjalbert et al., 2003). An important role for the Trx system in resistance against H_2_O_2_ has also been reported for *D. discoideum* (Jeong et al., 2006) and *A. alternate* (Ma et al., 2018). Indeed, TrxR-S can reduce Trx that allows the reduction of oxidized proteins.

*TrxR-S* presented a similar profile to *gpx* and *gr* regarding mRNA levels, with a 6.3-fold increase in expression in Lp WT-infected amoebae at 6 h p.i. when compared to NI amoebae (Fig 2E). This increase in expression was significant 24 h p.i. in amoebae infected with Lp WT compared to those infected with Lp Δ*dotA.* In addition, a transcriptomic analysis on *A. castellanii* infected by Lp WT revealed the up-regulation of this gene compared with amoebae infected with Lp Δ*dotA* (Li et al., 2020). Regarding the TrxR-S substrate, *i.e.* Trx, no increase in transcription was noted following an infection in our study. However, numerous genes are known to encode proteins with potential thioredoxin activity in *A. castellanii* (Clarke et al., 2013), and at least two thioredoxins were up-regulated during infection with *L. pneumophila* (Li et al., 2020).

For *trxR-L* (Fig 2G), in *A. castellanii*, the corresponding enzyme was considered as not being involved in oxidative defense (Leitsch et al., 2021). The decrease of *trxR-L* transcription at 6 h p.i. by Lp WT is probably due to the involvement of TrxR in reducing a wide range of substrates and not only Trx (Rigobello and Bindoli, 2010).

For *prx* (Fig 2H), amoebae infected with Lp WT or Lp Δ*dotA,* and amoebae exposed to H_2_O_2_ were down-regulated (around two-fold-change) compared to uninfected amoebae. In *A. castellanii*, this gene was considered to encode a protein not involved in oxidative defense (Leitsch et al., 2021). Among *prx* genes, only one might be responsible for ROS scavenging (Leitsch et al., 2021). Nevertheless, no up-regulation was noted for *prx* genes in *A. castellanii* infected with Lp WT, on the contrary, the set was mostly down-regulated (Li et al., 2020). Even if a previous study has reported an increased expression of *prx* in macrophages during *Brucella* infection, leading to a decrease in the production of ROS (Hu et al., 2019), it would appear that Prx is not the preferred choice for reducing H_2_O_2_ during *L. pneumophila* infection of *A. castellanii*.

Finally, for *sod*, amoebae infected with Lp WT or Lp Δ*dotA,* and amoebae exposed to H_2_O_2_ were significantly down-regulated (around two-fold-change) compared to uninfected amoebae (Fig 2I). The decrease observed in our study for *sod* transcripts under oxidative stress is in contradiction with a previous study of *A. castellanii* exposed to H_2_O_2_ which has shown a significant increase in the amount of *sod* transcripts and proteins (Motavallihaghi et al., 2022). This study has likely targeted a different class of SOD. Indeed, three various classes exist: classes one and three are Cu/Zn-SODs and class two is a Fe/Mn-SOD (Frye et al., 2022). Unfortunately, even if *A. castellanii* genome displays Cu/Zn and Fe/Mn SODs, our work focused on only one Fe/Mn-SOD, which represents the class two enzyme present in the mitochondrial matrix. Previous studies reported that Fe/Mn-SOD was not inducible under oxidative stress in *C. albicans*, or was strongly downregulated until 24 h p.i. in human foreskin fibroblasts infected with *Anncaliia algerae* (Enjalbert et al., 2003; Panek et al., 2014). Interestingly, no up-regulation was noted for *sod* genes in *A. castellanii* infected with Lp WT (Li et al., 2020).

Altogether, our results could indicate that the host GSH system expression is increased by *L. pneumophila* through its T4SS. A possible activation of the Trx system cannot be ruled out given the results obtained for TrxR-S. One hypothesis might be that *L. pneumophila* could use the glutathione system preferentially to reduce H_2_O_2_ and the Trx system to reduce oxidized proteins.

### 3.3 *L. pneumophila* infection modulates *A. castellanii* metabolome

**Fig. 3:**
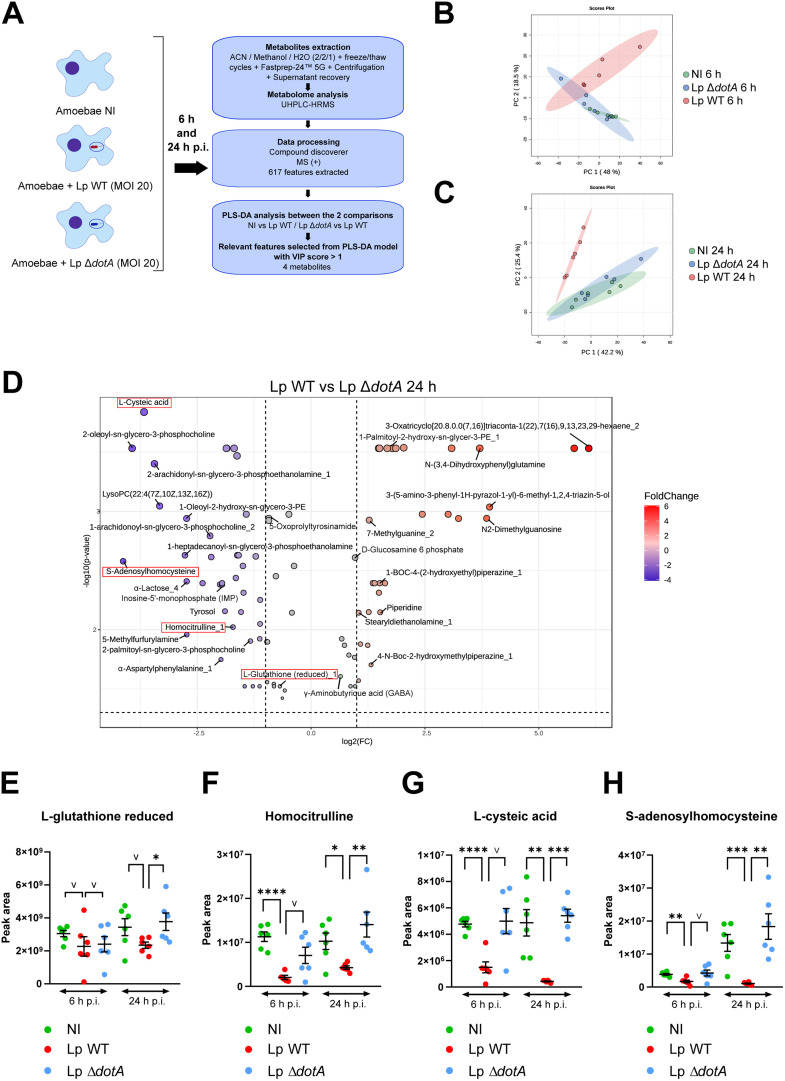
***Legionella pneumophila* disrupts *A. castellanii* metabolome.** (A) Schematic diagram of the experimental procedure used for the metabolomic analysis of non-infected *A. castellanii* (NI) or infected with *L. pneumophila* wild-type (Lp WT) or *dotA* deficient (Lp Δ*dotA*) at 6 or 24 h p.i. (MOI=20, n=6 per group). (B) PCA score plot generated using MetaboAnalyst 5.0 from metabolomic data at 6 h p.i.. (C) PCA score plot at 24 h p.i.. (D) Volcano plot of metabolites with a VIP score > 1 and p-value < 0.05 of FDR T-test for the Lp WT *vs* Lp Δ*dotA* comparison, the 4 metabolites selected for this study are framed in red. Box-plots based on peak area of (E) glutathione reduced, (F) homocitrulline, (G) L-cysteic acid and (H) S-adenosylhomocysteine selected from the PLS-DA model with a VIP score > 1 at 6 or 24 h p.i.. * p < 0.05; ** p < 0.01; *** p < 0.001; **** p < 0.0001 and v: indicative representation even if VIP score < 1.

To verify the putative enzymatic activity of antioxidant defense proteins consistent with the increase in transcripts at 6 h p.i., a non-targeted metabolomic analysis was carried out following infection of *A. castellanii* with *L. pneumophila* at 6 h and 24 h p.i. (Fig. 3A, Supplementary_data_2). A soft lysis of the amoebae followed by centrifugation to remove the bacteria from the samples was assessed prior to the study, to maximize our chances of observing the host metabolome exclusively. A first clustering analysis using a PCA based on the intensities of the 617 metabolites with a coefficient of variation ≤ 30 was carried out between the different conditions at 6 h and 24 h p.i. (Fig. 3B and C respectively). The PCA showed NI and Lp Δ*dotA* at 6 h and 24 h presented similar metabolite profiles. This is justified by the fact that Lp Δ*dotA* lacks a functional T4SS, rendering it incapable of multiplying within *A. castellanii* (Bandyopadhyay et al., 2007). The metabolomic profile of amoebae infected with Lp WT at 6h and 24h differs from that of NI and Lp Δ*dotA*, potentially due to the influence of T4SS effectors.

A second clustering approach using heatmap was also reported at 6 h p.i. (Fig. S3A) and at 24 h p.i. (Fig. S3B) confirming the previous observation. However, we also noted that NI, Lp Δ*dotA*, and Lp WT showed higher variability in metabolites profile between the replicates at 6 h p.i. compared to 24 h. It could be due to the low impact of Lp WT effectors on host metabolites at the early stage of the infection process. The metabolomic profile of amoebae, together with more bacteria associated with amoebae at 24h p.i. (Fig S1A and Fig. S3), lead us to think that 24 h p.i. was the best time to observe metabolic changes. In addition, no significant impact was reported on amoebae number and viability (Fig. S1B). We also chose to focus mainly on Lp WT *vs* Lp Δ*dotA* comparison.

PLS-DA, at 24 h p.i., enabled the selection of 275 out of 617 features based on variable importance in projection (VIP) score > 1. Unknown features were removed and 97 had a *p*-value < 0.05 of an FDR T-test (Fig 3D, Supplementary_data_3). A dozen lipids were in reduced abundance in Lp WT-infected amoebae than in those infected by Lp Δ*dotA*. which is consistent with a recent study showing that *L. pneumophila* exploits macrophage cell fatty acids to promote expansion of the replication vacuole and bacteria growth (Ghosh et al., 2024). α-Lactose was also reported to be present in smaller amounts, which is interesting since, to our knowledge, no study has reported a link between this carbohydrate and infection by *L. pneumophila*. Inosine-5′-monophosphate (IMP) quantity decreased in amoebae infected with *L. pneumophila.* This result is noteworthy given the critical role of IMP for nucleotide synthesis. This decrease may indicate a detour of this metabolite from the host to ensure the bacterium's nucleotides requirements during its multiplication. In our dataset, 2-deoxy-scyllo-inosose was decreased how much it was decreased in Lp WT-infected amoebae. 2-deoxy-scyllo-inosose synthase uses glucose-6-phosphate as a substrate for 2-deoxy-scyllo-inosose biosynthesis. D-glucose-6-phosphate could be isomerized by *L. pneumophila* to form D-fructose-6-phosphate and the glucosamine-6-phosphate synthase could use D-fructose-6-phosphate and L-glutamine to produce L-glutamate and D-glucosamine-6-phosphate (Floquet et al., 2009), which is found in greater quantities in Lp WT-infected amoebae. We could speculate that the bacterium hijacks the host's metabolism avoiding 2-Deoxy-scyllo-inosose biosynthesis to supply the D-glucosamine-6-phosphate that makes up peptidoglycan as it has already been reported with glutamine metabolism during *Chlamydia trachomatis* infection of endothelial cells (Rajeeve et al., 2020). γ-aminobutyric acid (GABA) was present in greater quantities in amoebae infected with Lp WT, few studies have investigated the role of this amino acid in amoebae, but it appears to be linked to the differentiation process in *Dictyostelum discoideum* (Anjard and Loomis, 2006). However, it is difficult to speculate on its role in the infection as a recent study has shown that GABA enhances autophagy, phagosomal maturation, and antimicrobial responses against *Mycobacterium tuberculosis* (Kim et al., 2018). Interestingly, four features have been linked to antioxidant defenses: L-glutathione reduced, homocitrulline, L-cysteic acid, and S-adenosylhomocysteine. At 24 h p.i., a significant decrease in all four metabolite abundances was observed in Lp WT-infected amoebae when compared to Lp Δ*dotA*-infected or NI amoebae (Fig. 3E, F, G, and H). The reduction observed at 24 h p.i. was at least 25 % for L-glutathione reduced (Fig 3E), 60 % for homocitrulline (Fig 3F), 93 % for L-cysteic acid (Fig 3G) and 92 % for S-adenosylhomocysteine (Fig. 3H). Indeed, we have shown that the Δ*dotA* mutant was not able to resist amoeba digestion and was no longer present intracellularly at 24h p.i. (Fig. S1A), suggesting that our results observed in amoebae infected with Lp WT are T4SS-dependent. We also note that a significant decrease of homocitrulline, L-cysteic acid, and S-adenosylhomocysteine was observed at 6 h p.i. in amoebae infected with Lp WT.

L-glutathione reduced (GSH) is an antioxidant tripeptide used as a substrate of Gpx to detoxify H_2_O_2_ within the cell. It is known that some bacteria can utilize host GSH for their benefit during infection. Indeed, *Francisella tularensis* may sense and steal GSH from the host as a cysteine source (van Hoek et al., 2024). Another study has highlighted ABC transporter substrate-binding protein GshT from *Staphylococcus pyogenes* as a key component of the glutathione salvage pathway from the host to be used in antioxidant defense (Brouwer et al., 2022). Normally, GSH is about 100-fold more abundant than GSSG in cells (Cassier-Chauvat et al., 2023), making the decrease observed here very interesting. Moreover, we reported a greater increase in *gpx* than *gr* transcripts at 6 h p.i. following infection by Lp WT when compared to the two other conditions. We hypothesize that reduced glutathione was oxidized more rapidly than reduced to ensure the observed reduction in ROS at 24 h p.i. (Fig. 2A), this could be a bacterial strategy to decrease ROS and allow the intracellular replication of *L. pneumophila* WT.

Homocitrulline can be produced through the carbamylation of lysine residues in proteins. This process involves cyanate, which originates from the spontaneous decomposition of urea or the action of myeloperoxidase on thiocyanate in the presence of H₂O₂ (Pireaux et al., 2021). The decrease in homocitrulline observed in amoebae infected with *L. pneumophila* WT at 24 h p.i. could also be explained by a drop in cyanate production as ROS levels are reduced. In the mitochondria, carbamyl phosphate may bind lysine forming homocitrulline (Martinelli et al., 2015). In this context, a previous study has shown the implication of homocitrulline in the increase of H_2_O_2_ production and the decrease of GSH levels (Zanatta et al., 2013). Based on this observation, the reduction of host homocitrulline may serve as a bacterial strategy to evade oxidative stress within the host cell.

The detection of L-cysteic acid in amoeba is interesting, given its rare mention in the existing literature. This metabolite is part of the taurine biosynthesis pathway, and more specifically the Serine/Sulfate pathway, found notably in microalgae (Tevatia et al., 2015). As a precursor of taurine, this decrease of host L-cysteic acid could be the reflection of increased taurine production by the cysteine sulfinic acid decarboxylase and glutamate decarboxylase and it would be interesting to do a targeted metabolic analysis of taurine to confirm this hypothesis. The hijacking of taurine from the host could be another strategy used by bacteria to ensure an antioxidant state in the host.

Finally, S-adenosylhomocysteine is a well-known intermediate of the transsulfuration pathway leading to the production of cysteine from methionine (Sbodio et al., 2019). It is known that *L. pneumophila*, to obtain host amino acids notably cysteine, hijacks the conserved polyubiquitination and proteasomal degradation machinery (Price et al., 2014, 2011). Our results suggest another strategy employed by *L. pneumophila* to obtain cysteine by scavenging the cysteine synthesized by the host. Indeed, the decrease of host S-adenosylhomocysteine could be the reflection of increased cysteine synthesis. Another hypothesis suggests that the decrease in this metabolite abundance during *L. pneumophila* infection could be attributed to the synthesis of glutathione, as cysteine is one of the three constituent amino acids. This hypothesis aligns with the increase in D-glucosamine-6-phosphate involving an increase in L-glutamate and the results obtained for GS expression, which was upregulated at 6 h p.i. in amoebae infected with *L. pneumophila* WT.

Altogether, these results support the involvement of the glutathione system during infection and other possible strategies used as taurine synthesis or homocitrulline decrease. It paves the way for new avenues of research, providing clues to other possible strategies used by the bacterium to maintain an antioxidant state within its host, that is probably more conducive to its intracellular multiplication.

### 3.4 Exposure to ROS could promote intracellular multiplication of *L. pneumophila*

**Fig. 4:**
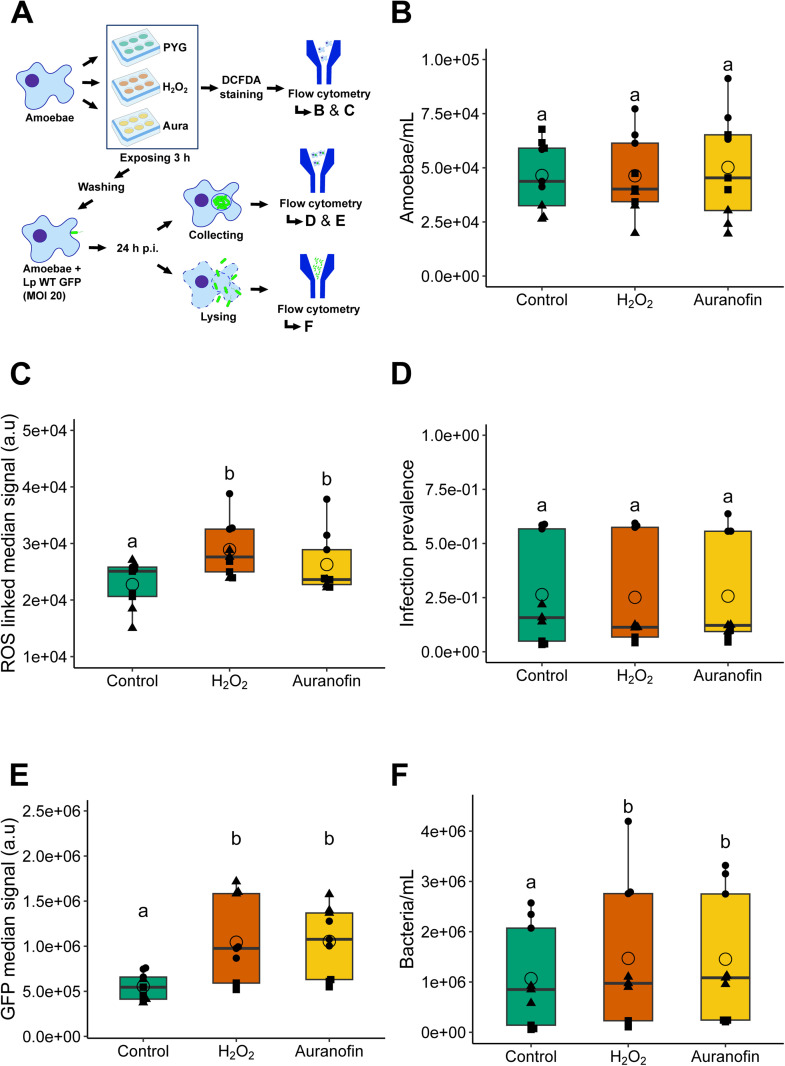
**Pro-oxidizing pre-treatment of *A. castellanii* may enhance the intracellular multiplication of *L. pneumophila*.** (A) Schematic diagram of the experimental procedure used with untreated amoebae (Control) or 250 µM H_2_O_2_ (H_2_O_2_) and 5 µM Auranofin (Aura) pretreated amoebae before infecting them with *L. pneumophila* WT GFP (Lp WT GFP) (B) Number of amoebae per mL after 3 h pretreatment (C) CM-H_2_DCFDA corresponding median fluorescence signal in arbitrary units (a.u.) after pretreatment (D) Infection prevalence based on GFP positive amoebae at 24 h p.i.. (E) GFP median fluorescence signal (a.u) of infected amoebae at 24 h p.i.. (F). Number of GFP bacteria per mL at 24 h p.i. from lysed amoebae. Box plots are based on at least three independent experiments done in triplicate. Each symbol represents data from a biological replicate. Two points not connected by the same letter are significantly different.

Starting from the hypothesis that *L. pneumophila* could use the antioxidant defenses of the amoeba to its advantage during the infection, we pretreated amoebae before infection with two molecules, H_2_O_2_ and auranofin, which is a pro-oxidative agent inhibiting the thioredoxin reductase system, to induce oxidative stress. In addition, both are known to increase the expression of several genes encoding enzymes of involved in antioxidant defenses (Köhsler et al., 2022). The aim was to potentially trigger host antioxidant defenses before infection, through the induction of ROS production, to evaluate the effect on the infectious cycle of the bacterium. For this purpose, we checked the ROS level of amoebae after exposure by H_2_DCFDA labeling measured with flow cytometry, and then a fluorescent bacterium (Lp WT GFP) was used to infect amoebae according to a protocol detailed in Fig. 4A. Preliminary assays allowed us to determine the most favorable conditions to have an effect in amoebae, without affecting their number and viability. For this reason, amoebae were pre-exposed during 3 h to 250 µM of H_2_O_2_ and 5 µM of auranofin. As expected, the treatments had no impact on amoebae number compared with non-treated amoebae (Fig. 4B) but increased significantly ROS-associated median signal of H_2_DCFDA fluorescence (Fig. 4C). Indeed, H_2_O_2_ and auranofin pre-treatment increased significantly the signal by about 30% and 15% respectively.

We next infected the amoeba with Lp WT or Lp Δ*dotA* after 3 h pre-treatment with H_2_O_2_ or auranofin. At 24 h p.i. the amoebae were harvested and analyzed by flow cytometry. No variation in infection prevalence was observed between the conditions (Fig. 4D), suggesting that pre-treatment does not increase the number of infected amoebae. Still, a significant increase of the GFP signal was noticed in infected amoebae treated with H_2_O_2_ and auranofin (Fig 4E). To confirm this result, we have lysed amoebae and quantified the number of intracellular GFP bacteria by flow cytometry. The number of intracellular bacteria was 36% and 28% higher when pre-treated with H_2_O_2_ and auranofin respectively (Fig. 4F). Taken together, these results suggest that amoebae treatment with H_2_O_2_ or auranofin could promote *L. pneumophila* intracellular multiplication. However, CFU counting on agar plates did not confirm this observation (data not shown). It is important to note that counting *L. pneumophila* on agar plates cannot be directly compared to counting by flow cytometry, as cultivability represents only a subset of the bacterial population and may differ significantly from total counts. If we did not observe an identical result with cytometry and CFU, we cannot exclude the possibility that pre-treatments influenced the intracellular multiplication of the bacteria, and extended studies are required to clarify whether pro-oxidative treatments could promote *L. pneumophila* intracellular multiplication. However, data from previous studies suggest that treatment with pro-oxidative compound could upregulate amoeba antioxidant defense transcripts. Indeed, it was already shown that auranofin-treated amoebae presented upregulated GSH system enzymes, while expression of both TrxRs was down-regulated (Köhsler et al., 2022). The same study showed that H_2_O_2_-treated amoebae showed a preferential increase in the Trx system, as we observed at 6h p.i. Thus, potential activation of defense systems by exposure to H_2_O_2_ or auranofin before infection could promote *L. pneumophila* multiplication.

**Fig. 5:**
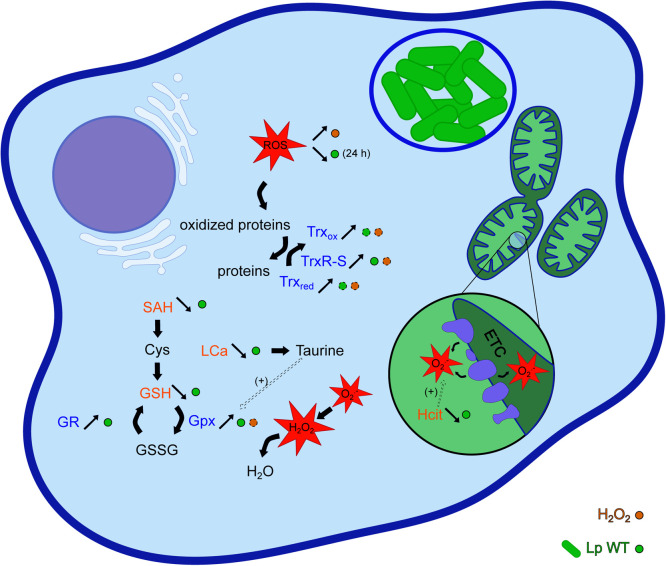
**Hypothetical overview of the effect of *L. pneumophila* on ROS levels and antioxidant defenses of *A. castellanii.*** The dashed arrows or circles indicate effects depicted in other studies. ETC: Electron transport chain, Gpx: Glutathione peroxidase, GR: Glutathione reductase, GSH and GSSH: Glutathione reduced and oxidized respectively, Hcyt: Homocitrulline, Lca: L-Cysteic acid, SAH: S-Adenosylhomocysteine, Trx_red_ and Trx_ox_: Thioredoxin reduced and oxidized respectively, TrxR-S: low molecular weight Thioredoxin reductase. Metabolites reported in the non-targeted metabolomic analysis are written in orange and the corresponding enzymes whose genes expression has been studied using RT-qPCR are written in dark blue.

Fig. 5 summarizes our observations about the two approaches of RT-qPCR and non-targeted metabolomics.

Our results suggest that *L. pneumophila,* through its T4SS, could increase the transcription of both *gpx* and *gr* which could increase the quantity of corresponding enzymes. On the other hand, the observed increase in *trxR-S* transcription could be linked to those of the *trx* which would result in potential use of the Trx system to reduce oxidized proteins. This strategy of using the host's antioxidant defenses to the bacterium's advantage is a theory that can be corroborated by the results of other publications. Indeed, it was first shown that *Mycoplasma hyopneumoniae* induces an increase in the antioxidant defenses of host epithelial cells upon infection of the latter (Mucha et al., 2020), and *Coxiella burnetii* uses a Dot/Icm effector called Mitochondrial Coxiella effector protein F (MceF), which localizes to the mitochondria and recruits the antioxidant protein glutathione peroxidase 4 (Gpx4) from the macrophage to prevent oxidative stress-induced cell death (Loterio et al., 2023).

We propose that the observed drop in ROS at 24 h p.i. may result from bacterial enhancement of the host antioxidant defenses, during its intracellular multiplication. This augmentation of host antioxidant defenses could substitute those of *L. pneumophila*, such as alkylhydroperoxydase, used during late stage of *A. castellanii* infection (Quan et al., 2020). In the early stages of infection, *L. pneumophila* suppresses ROS production by infected U937 cells through its T4SS due to the failure of NADPH oxidase activation through inhibition of p47phox recruitment to phagosomes containing the bacterium (Harada et al., 2007), but this does not justify the decrease in ROS observed at 24 h p.i. (Kajiwara et al., 2018). In the case of the amoeba, we would first have to confirm this inhibition of NADPH oxidase, which is probable, as our results do not indicate any increase in ROS at early stages (Fig 1A). This strategy is similar to that observed with *Coxiella burnetii* that also target the NADPH oxidase to promote its intracellular replication (Siemsen et al., 2009). We also know that *L. pneumophila* infection led to a modulation of mitochondrial dynamics in macrophages thanks to MitF that promotes a Warburg-like phenotype in macrophages favorizing the glycolysis and leading to a decrease in OXPHOS and potentially ROS by-product (Escoll et al., 2017). A previous study using metformin to increase mitochondrial ROS resulting in a replication defect in *L. pneumophila* highlighted the importance for the bacterium of maintaining mitochondrial ROS at control levels to ensure the smooth running of its replicative cycle (Kajiwara et al., 2018). In particular, this maintenance could be justified by the metabolic switch, with the establishment of a Warburg-like effect through *L. pneumophila*'s ability to control macrophage mitochondria (Escoll et al., 2017).

Our results show that in addition to the strategies already described, *L. pneumophila* WT could increase the transcription of genes encoding Gpx and GR, resulting in increased H_2_O_2_ detoxification and reduced ROS within its natural host at 24 h p.i.

Just as a precedent study has previously demonstrated the importance of non-increased mitochondrial ROS for the proper functioning of the cell cycle, our results ask the question of the importance of maintaining ROS globality at sub-normal concentrations for the bacteria. The use of ROS inhibitors or activators would appear to be an interesting avenue of research for future experiments.

Finally, *L. pneumophila* may use other strategies to keep the host ROS low, potentially increasing the synthesis of glutathione by the transsulfuration pathway, increasing the synthesis of taurine by the Serine/Sulfate pathway, and decreasing homocitrulline known to increase ROS levels. Influencing these metabolic pathways with enzymatic inhibitors or activators is also an interesting avenue to a better understanding of the role of oxidative stress in the infectious cycle of *L. pneumophila*.

## 4 Conclusion

In conclusion, our study showed that *L. pneumophila*, through its T4SS, upregulates transcripts associated with the glutathione system in its host *A. castellanii* at 6 h p.i., and potentially influences the thioredoxin system as well. Until now, it was known that *L. pneumophila* could prevent the recruitment of NADPH oxidase in the early phase of infection, thereby suppressing the host's defensive production of ROS. It was also known that *L. pneumophila* decreased overall host ROS at 24 h p.i. in macrophages, without however explaining this decrease. In addition to confirming the decrease in ROS in amoebae, our study provides clues as to the ability of *L. pneumophila* to use the antioxidant defenses of its amoebic host, as well as the latter's metabolism, to create an antioxidant environment. The challenge now is whether this environment is necessary for bacterial multiplication. Targeting this bacterial manipulation of the host's antioxidant defenses could be a promising approach to limit the multiplication of the bacterium in the environment.

## CRediT authorship contribution statement

**Alban Hay**: conceptualization, methodology, validation, formal analysis, investigation, writing - original draft, visualization. **Willy Aucher**: methodology, investigation, resources, writing – review & editing. **Romain Pigeault**: formal analysis, resources, writing – review & editing, visualization. **Joanne Bertaux**: methodology, resources, writing – review & editing. **Alexandre Crépin**: methodology, resources, writing – review & editing. **Quentin Blancart Remaury**: methodology, resources, writing – review & editing. **Yann Héchard**: writing – review & editing, supervision. **Ascel Samba-Louaka**: conceptualization, methodology, writing – review & editing, supervision, project administration. **Romain Villéger**: conceptualization, methodology, investigation, writing – review & editing, supervision, project administration.

## Funding

Alban Hay's thesis was funded by the University of Poitiers.

## Declaration of competing interest

The authors declare that they have no known competing financial interests or personal relationships that could have appeared to influence the work reported in this paper.

## Data Availability

Data will be made available on request.

## Appendix Supplementary materials

**Fig. S1. *L. pneumophila* multiplication in *A. castellanii* and amoebae proliferation upon *L. pneumophila* infection.***A. castellanii* non-infected (NI) or infected with *L. pneumophila* WT (Lp WT) and ∆*dotA* (Lp ∆*dotA)* were plated. At 6 and 24 h post-infection, cells were harvested and counted (B). A part of them was lysed and the released bacteria were plated for Colony-Forming Units (CFU) enumeration (A). Results of three independent experiments, the error bars represent the standard error of the mean. ns = non-significant, * p < 0.05. Image, application 1

**Fig. S2. Workflow used on Compound Discoverer 3.3**. Image, application 2

**Fig. S3. One-way hierarchical clustering heatmaps.** Relative metabolites abundance at 6 h p.i. (A) and 24 h p.i. (B). Image, application 3

Image, application 4

Image, application 5

Image, application 6
