# Supplementary figures and images for: Legionella pneumophila subverts the antioxidant defenses of its amoeba host Acanthamoeba castellanii
Source: Curr Res Microb Sci. 2025 Jan 7;8:100338. doi: 10.1016/j.crmicr.2024.100338 (PMC11772960; doi:10.1016/j.crmicr.2024.100338)

**A**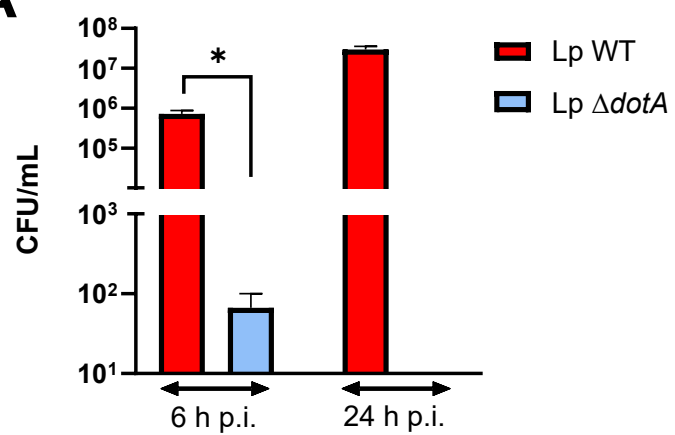**B**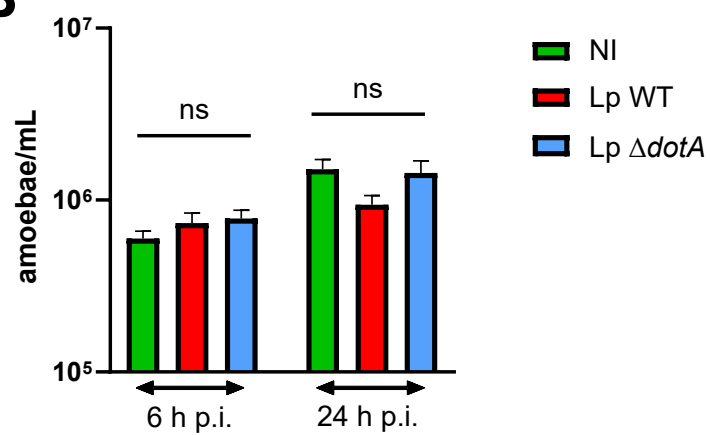

Supplement: Supplementary file 1 — Fig. S1. L. pneumophila multiplication in A. castellanii and amoebae proliferation upon L. pneumophila infection.A. castellanii non-infected (NI) or infected with L. pneumophila WT (Lp WT) and ∆dotA (Lp ∆dotA) were plated. At 6 and 24 h post-infection, cells were harvested and counted (B). A part of them was lysed and the released bacteria were plated for Colony-Forming Units (CFU) enumeration (A). Results of three independent experiments, the error bars represent the standard error of the mean. ns = non-significant, * p < 0.05. [file mmc1.pdf]

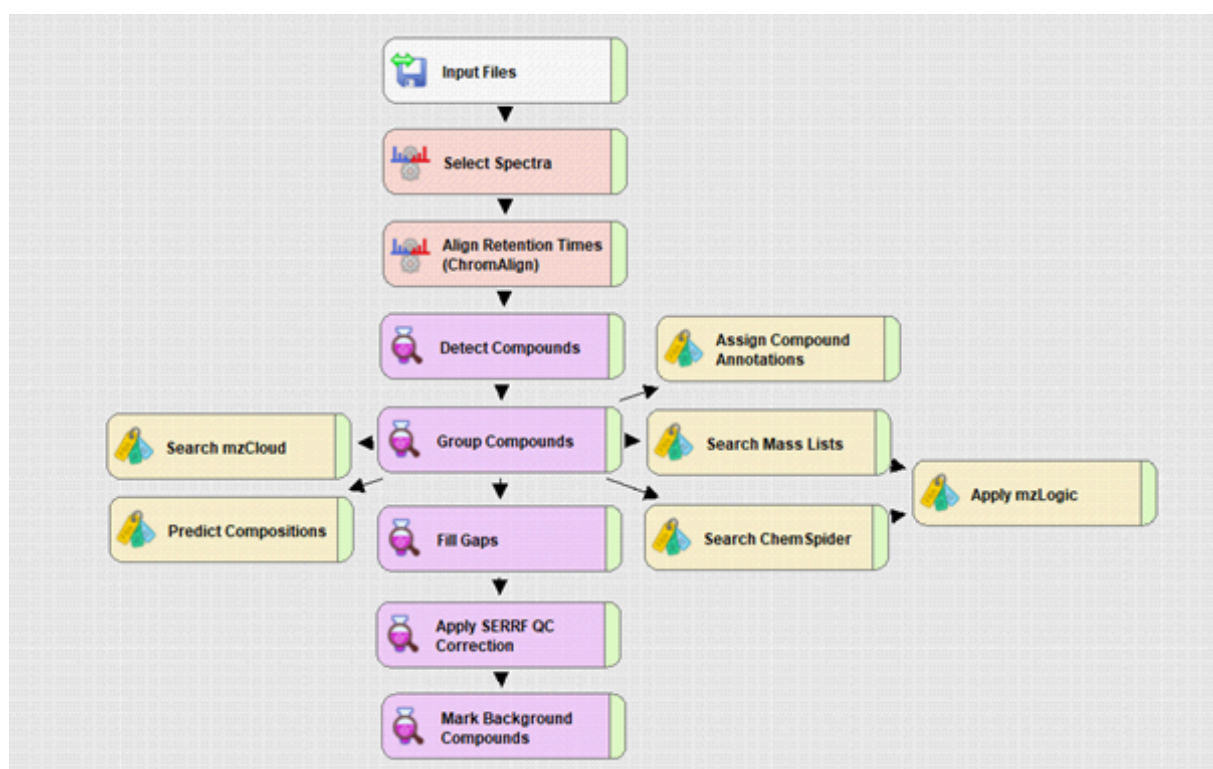

Supplement: Supplementary file 2 — Fig. S2. Workflow used on Compound Discoverer 3.3. [file mmc2.pdf]

A

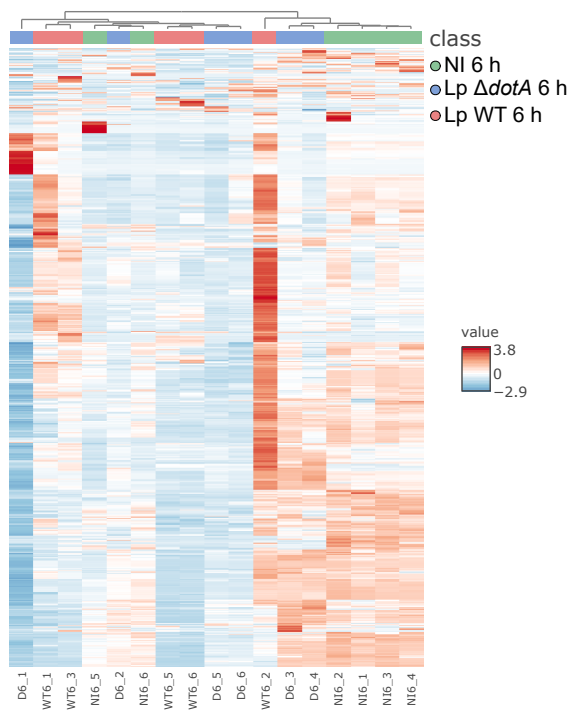

B

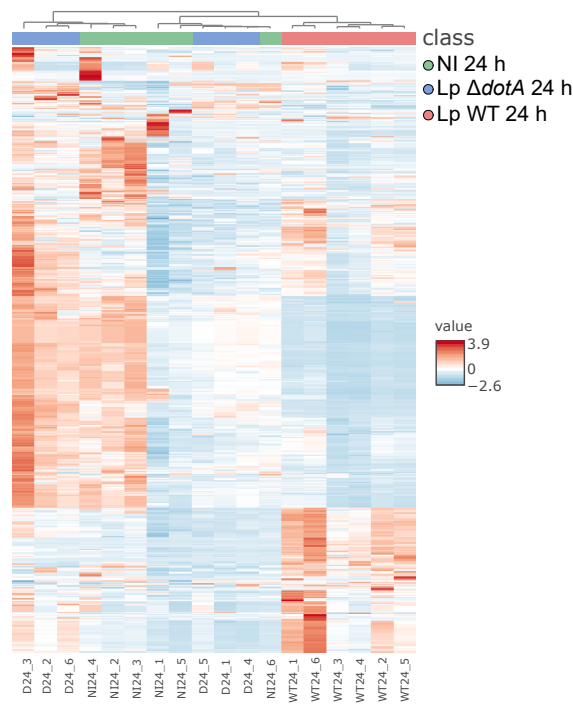

Supplement: Supplementary file 3 — Fig. S3. One-way hierarchical clustering heatmaps. Relative metabolites abundance at 6 h p.i. (A) and 24 h p.i. (B). [file mmc3.pdf]
